# In-Depth Transcriptome Analysis Reveals Novel TARs and Prevalent Antisense Transcription in Human Cell Lines

**DOI:** 10.1371/journal.pone.0009762

**Published:** 2010-03-25

**Authors:** Daniel Klevebring, Magnus Bjursell, Olof Emanuelsson, Joakim Lundeberg

**Affiliations:** Division of Gene Technology, School of Biotechnology, AlbaNova University Center, Royal Institute of Technology, Stockholm, Sweden; George Washington University, United States of America

## Abstract

Several recent studies have indicated that transcription is pervasive in regions outside of protein coding genes and that short antisense transcripts can originate from the promoter and terminator regions of genes. Here we investigate transcription of fragments longer than 200 nucleotides, focusing on antisense transcription for known protein coding genes and intergenic transcription. We find that roughly 12% to 16% of all reads that originate from promoter and terminator regions, respectively, map antisense to the gene in question. Furthermore, we detect a high number of novel transcriptionally active regions (TARs) that are generally expressed at a lower level than protein coding genes. We find that the correlation between RNA-seq data and microarray data is dependent on the gene length, with longer genes showing a better correlation. We detect high antisense transcriptional activity from promoter, terminator and intron regions of protein-coding genes and identify a vast number of previously unidentified TARs, including putative novel *EGFR* transcripts. This shows that in-depth analysis of the transcriptome using RNA-seq is a valuable tool for understanding complex transcriptional events. Furthermore, the development of new algorithms for estimation of gene expression from RNA-seq data is necessary to minimize length bias.

## Introduction

Less than 2% of the human genome encodes for proteins, yet a large fraction, recently estimated to 60% to 90% of the genome can be transcribed [1]. The functions of the majority of these novel uncharacterized transcriptionally active regions (TARs) are currently unknown, but they are believed to be of regulatory importance. For example, Ebisuya and colleagues showed that “transcriptional ripples” can propagate along the genome and mediate regulation of genes several tens of kilobases away [2].

Several studies [3] have shown that antisense transcription is prevalent and likely to have a regulatory function. Studies indicate that 20% to 90% of all human protein-coding genes can generate transcripts with potential to form sense-antisense pairs [4]–[6] and that these generally are arranged in a tail-to-tail pattern. Recently, short fragments of RNA have been detected in the antisense direction in regions just upstream protein-coding genes [7]–[9].

In parallel to experimental discovery of regulatory RNAs, computational methods are being developed to identify conserved structural RNA elements likely to be involved in transcriptional and translational control [10]. These approaches aim to make in silico predictions of regulatory sites in the human genome that can be validated by the on-going massive transcriptome sequencing (RNA-Seq) efforts on cells, tissues and organs [11], however, more development is needed to make these algorithms more accurate and efficient.

In this study, we use massive DNA sequencing to investigate RNA longer than 200 nucleotides from three human cancer cell lines. We show that approximately 20% of all protein-coding genes have antisense transcription coupled to them and that antisense transcription is prevalent in introns.

## Results

### Experimental outline

In this study we investigate the transcriptome of three cell lines, A431, U-2 OS and U251, by applying the massive SOLiD DNA sequencing technology facilitating sense/antisense identification of reads. The cell lines were chosen to represent three different lineages; epithelial, mesenchymal and glia cells. A total of 10 to 15 million high quality 50-basepair reads were obtained for each cell line. The reads were mapped onto the human reference genome (hg18), after which reads were aggregated for each gene. An expression value was calculated based on the number of reads per kilobase gene and million reads in each sample (RPKM) [12]. Analysis of the gene expression pattern demonstrated that 66% to 69% of all genes are expressed in each cell line of which 85% to 88% were shared for all three cell lines (figure S1).

### Comparison of RNA-seq and microarray gene expression data

**Figure 1:**
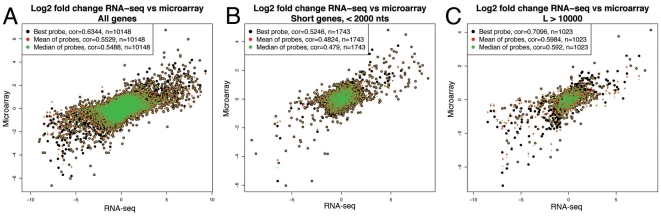
Scatter plot of RNA-seq (x-axis) versus microarray log2(fold change) (y-axis) for all protein coding genes (A), genes shorter than 2000 nts (B) and genes longer than 10 000 nts (C). Colors dots indicate the most similar microarray probe (black), the mean probe ratio (red) and median ratio (green). Longer genes correlate better with microarray data than short genes.

To validate the results obtained from RNA-seq, we compared the data to gene expression data from the A431 and U251 cell lines obtained using microarrays (no data was available for U-2 OS). Since the microarray platform only generates relative expression values, the correlation between the RNA-seq data and the microarray data was calculated using the log2 value of the ratio between A431 and U251, which in the RNA-seq case yields one value per Ensembl-gene. Since one gene can be represented by several microarray probes, we used three different methods to convert these to a single value that could be compared to the RNA-seq data (mean, median and best probe, see Materials and Methods for details). The Spearman correlation was determined to 0.55, 0.55 and 0.64 for the three methods respectively, values in the same range as those described earlier [13]. Oshlack and Wakefield recently showed that the variance estimation of the RPKM measure is dependent on the gene length [14]. Thus, we hypothesized that the correlation between microarray data and RNA-seq data would share this dependence, since the log2-fold change in RNA-seq will have lower variance for longer genes that for shorter genes. This assumption turned out to be correct; for genes shorter than 2000 bases, the correlation was 0.48 to 0.52 depending on method, while for genes longer than 10 kb, this range was 0.59 to 0.71 (figure 1B–C and figure S2).

### Investigation of antisense expression

**Figure 2:**
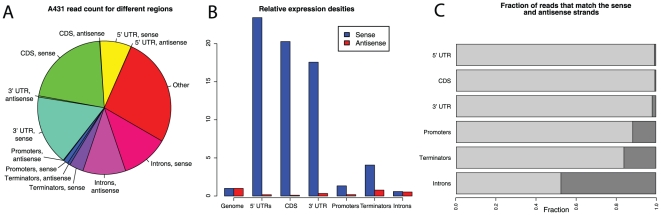
(A) Pie chart describing read mappings to different genomic regions. Almost half of all reads map to known genes (5 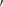 UTRs, CDS and 3 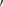 UTRs). A large fraction of reads map to regions outside promoters, known genes or terminator regions (red). A very low fraction of all reads map to antisense to protein coding genes. (B) Relative expression densities in different genomics regions. For the antisense strand, a small increase can be seen in promoter and terminator regions. Error bars are one standard deviation calculated across all three samples. (C) Fraction of reads that map to different regions in the genome. In introns, roughly half of all reads map to the antisense strand. Sense in light gray, antisense in dark gray.

The vast majority of all reads originate from the sense strand of protein-coding genes (figure 2A and figure S3). A large fraction of the reads also originate from the introns of protein-coding genes, but when normalized to the length of the introns, the relative expression levels of introns are very low (figure 2B and figure S3, S4, S5). We also note that many reads map to regions distant from protein-coding genes (here denoted as “Other”), which to some extent can be expected since this includes many long non-protein-coding genes. Previous studies have described a class of short transcripts (20–90 nucleotides) that originate from the antisense strand in the promoter regions of genes [7]–[9], [15]. We investigated tag densities in promoter and terminator regions (defined as 1000 base pairs upstream and downstream of genes, respectively) and are unable to detect an increased density upstream of genes. This is expected since our extraction method does not capture fragments shorter than roughly 200 nucleotides. In the terminator regions, however, the relative antisense tag density is higher than that in exons and promoter regions (figure 2B and figure S3). This indicates that transcription of long RNAs in terminator regions could represent a regulatory mechanism for termination of transcription. We investigated the sense-to-antisense ratio for different regions of the genome. In protein-coding exons, 98% to 99.5% of the reads originate from the sense strand, indicating that antisense transcripts are present at very low levels (figure 2C and figure S3). Interestingly, the sense-to-antisense ratio is markedly increased for promoter and terminator regions. In promoter regions, about 12% of the reads originate from the antisense strand, and in terminator regions, the fraction is 16%. In introns, the corresponding number increases to approximately 50% (figure 2C and figure S3).

### Identification of novel TARs

**Figure 3:**
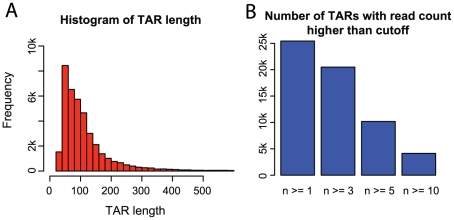
(A) Histogram of detected TAR lengths. Many regions appear as shorter than 200 base pairs which is likely caused by the fact that they are very lowly expressed. The RNA extraction method captures fragments longer than 200 nucleotides, indicating that the majority of the detected TARs are in fact longer than we detect. (B) Bar plot describing number of reads in the detected TARs. Roughly 5 000 of the 40 000 TARs are detected by more than 10 reads. Error bars are one standard deviation calculated across all three samples.

To identify novel TARs, we merged reads from all three cell lines and created clusters from overlapping and nearby reads. After subtraction of known genes and non-coding RNA genes, we identify approximately 40,000 novel TARs, of which most are short (figure 3A). In fact, only 1360 TARs are longer than 500 base pairs and only 508 are longer than 1000 base pairs. Expression values for all TARs were calculated using the same approach as for protein-coding genes. This showed that most TARs are lowly expressed and covered by few reads. In A431, only approximately 10% (4144 TARs) are detected by 10 reads or more (figure 3B). The corresponding number in protein-coding genes is 33%, but this number is likely biased by the fact that protein-coding genes are generally longer than the putative novel TARs (data not shown). The method for RNA extraction used in this study excludes fragments shorter than approximately 200 nucleotides. This suggests that the majority of TARs identified in this study are in fact 200 nucleotides or longer and that deeper sequencing is needed to cover the entire TARs in order to define their boundaries.

**Figure 4:**
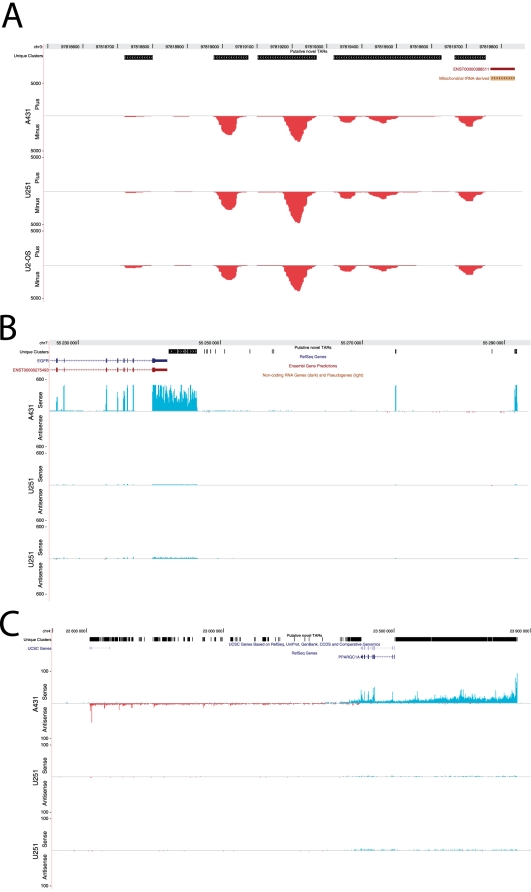
(A) Transcription downstream of a known tRNA pseudogene. Several TARs are detected in all three cell lines. The pseudogene itself has perfect identity to another region in the genome, so reads derived from is will not map unambiguously are are discarded. (B) Several TARs are detected downstream the gene EGFR. A group of them likely describe a prolonged 3 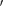 UTR. Two TARs further downstream could represent new exons. The gene and TARs are detected at higher levels in A431 than the other samples, which agrees with previous knowledge [16]. (C) Massive transcription from a region around PPARGC1A. Transcription is detected from the sense strand several hundred kilobases downstream of the gene, as well as from the antisense strand several hundred kilobases upstream in A431. Transcription from this region is almost completely shut off in U251 and U-2 OS.

Even though most novel TARs seem to be lowly expressed, we find a few interesting instances among these. Several clusters are detected downstream of a tRNA pseudogene on chromosome 3. We believe that this is the result of transcription which has been initiated upstream of the pseudogene and continues downstream (Figure 4A). The pseudogene itself has 100% sequence identity to another region in the genome (chr5:79,982,623–79,982,691), and since only reads that map uniquely to the genome were used in this analysis, this gene appears not to be expressed. Very high expression of EGFR is one of the hallmarks for the A431 cell line [16]. In figure 4B, a prolonged exon of the epidermal growth factor receptor (*EGFR*) is shown, along with two small clusters several tens of kilobases away. Whether or not the two small clusters are in fact novels exons remains to be investigated. In figure 4C, transcription is detected from both strands of a 1.3 Mb-region surrounding Peroxisome Proliferator-activated Receptor 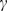 Coactivator-1 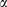 (*PPARGC1A*) on chromosome 4. Expression from this regions is detected at high levels in A-431 cells, but is almost completely shut off in U-2 OS and U-251 cells. This provides an intriguing example of complex transcription, and could indicate gene regulation through antisense transcript expression. This gene has been implicated in diabetes where lower expression has been linked to insulin resistance and DNA damage [17]. What functional role the antisense transcript plays remains to be elucidated.

## Discussion

In the current study we have investigated the transcriptional levels of three human cancer cell lines using RNA-seq. We show that the correlation between DNA microarray data and RNA-seq data depends on gene length, and that the reason for this is the increased precision in expression level measurements for longer genes due to the fact that a higher number of reads will map to longer genes than short, as described earlier [14]. In this study, this means that the correlation between DNA microarrays and RNA-seq ranges from approximately 0.48 to 0.71 depending on which gene length and microarray probe selection method is used. However, this points to a larger statistical issue when using RNA-seq data to assess differential expression, since long genes will bias for example lists of differentially expressed genes between samples, and thus influence the power of gene set enrichment analysis negatively. Future research in this area will certainly have to address this issue, for example by improved statistical methods or by limiting the analysis to reads mapping to the 3 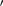 part of the gene with length equal to the shortest gene included in the analysis.

We show that for approximately 20% of all human protein-coding genes, there is at least weak antisense transcription to exonic sequences. We also show that many of the antisense signatures overlap between the investigated cell lines (figure S3b). During recent years, several studies have indicated that 20–90% of all human genes can generate antisense transcripts that can mediate regulation of the sense transcript [4]–[6]. Our study falls into the lower end of that interval, possibly indicating that deeper sequencing is required to investigate this phenomenon further. We also investigate antisense transcription in different regions of the genome. He and colleagues demonstrated that antisense transcription was prevalent upstream of transcription start sites, and Preker and colleagues showed that these transcripts are polyadenylated and short (20–90 nucleotides) [7], [18]. We do not identify such a pattern, which is likely explained by the fact that our study targets transcripts longer than 200 nucleotides. After clustering reads, we identify many novel TARs, most of which are shorter than 200 base pairs. This is likely due to the fact that they are generally lowly expressed, and a deeper sequencing of these samples would likely reveal the remaining parts of these TARs. Interestingly, we see approximately equal levels of transcription from both strands of introns of protein-coding genes. Non-protein coding intronic transcripts have been shown to be enriched in genes related to transcription regulation and interact with promoters to mediate regulation [19], [20].

The ENCODE project showed that transcription was frequent even outside of protein-coding genes [1], and with the recent emerge of new sequencing technologies, vast numbers of new transcriptionally active units have been detected. These TARs are situated in a non-random pattern along the chromosomes, indicating that they are not general background transcription. Some also show patterns of differential expression (figure 4). As more in-depth transcriptome studies deposit their data into publically available warehouses, such as Gene Expression Omnibus (http:www.ncbi.nlm.nih.gov/geo), more regions like these will likely be detected and characterized. It will be of great importance to functionally characterize these novel non-protein-coding transcripts and their potential role in gene regulation.

## Materials and Methods

A431, U-2 OS and U251 cells were grown as described earlier [21]. Cells were harvested and RNA was extracted using the RNeasy mini kit (Qiagen, Valencia, CA) following the manufacturer's instructions, and 15 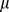 g of total RNA was used as input material for the SOLiD Whole Transcriptome kit (Applied Biosystems Inc., Foster City, CA) and 14 372 246, 10 547 681 and 11 449 673 reads (each 50 nucleotides) passed quality filters including filtering against adaptors. The reads were mapped to chromosomes 1–22, X and Y of the human genome (hg18) using Corona lite with default parameters (Life Technologies/Applied Biosystems).

### Comparison with microarray data

Two-color DNA microarray data for the cell lines A431 and U251 was provided by Gry *et al.*, and was pre-processed as described elsewhere [22]. The quality of the arrays has previously been addressed by comparison with MAQC data [23]. To allow for comparison to with RNA-seq data, RPKM expression levels were calculated for every Ensembl gene (http:www.ensembl.org) as described elsewhere [12], and a log2-fold change was calculated for the ratio A431 versus U251. Since one gene can be interrogated with several microarray probes, three different methods were used transform the microarray expression data to one value per gene; the mean of all probes, the median of all probes or the probe with the value closest to the RNA-seq data. We used Spearman's rho to quantify the correlation between the two platforms.

### Sense versus antisense expression regions

To investigate the sense and antisense expression in different genomic regions, we calculated the number of reads that map to each region of interest. Some regions (coding regions, introns, 5 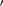 UTRs and 3 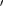 UTRs of protein-coding genes) were downloaded as BED-files from the UCSC table browser. Promoter and terminator regions were defined as 1 kb upstream or downstream of a protein-coding gene, respectively, similar to what has been used earlier [7]. If a neighbouring gene resides within the promoter or terminator region, the overlap with this gene was removed from the promoter or terminator region. For each region type, we calculated the expression density by counting number of reads that map entirely within the region type and normalized to the total length of the regions and the total amount of sense or antisense reads. This procedure yields one relative tag density value for each region type, sense and sample. We also calculated the sense-to-antisense ratio for each regions type and sample.

### Identification of novel transcriptionally active regions

To identify putative novel transcriptionally active regions, we clustered reads (using the online-version of Galaxy, [24] allowing for reads to be 15 bases apart and require at least three reads to be present to form a cluster. These first clusters were then merged across cell lines. We then subtracted clusters that overlap with known genes (as defined by Ensembl genes) as well as non-coding RNA genes (RNA genes, UCSC Genome Browser).

## Supporting Information

Figure S1 Overlap of sense and antisense expression between the cell lines.
(0.23 MB PDF)

Figure S2 Correlation to microarray data, binned per gene length in intervals of 2000 bps. See main text for discussion.
(11.70 MB TIF)

Figure S3 Information on read mappings for additional cell lines. (D, G) Fraction of reads mapping to different regions. (E, H) Relative tag density in different regions. (F, I) Fraction reads mapping to the sense and antisense strand for different regions. See main text for discussion.
(0.20 MB PDF)

Figure S4 Smooth scatterplots of log10(rpkm) between samples along with Spearman's rho correlation. The correlation is .87 to .88 between all samples. This indicates that most genes have similar levels across all samples.
(1.41 MB PDF)

Figure S5 Smooth scatterplots of log10(antisense-rpkm) between samples. Spearman's rho correlation coefficient is here slightly lower than that in the sense-case (supplementary figure S4). A reason for this could be that the majority of antisense transcripts are lowly expressed. It is also possible that these antisense transcripts have regulatory function and differ more than the bulk of mRNAs expressed in a cell.
(1.41 MB PDF)
