# Supplementary figures and images for: In-Depth Transcriptome Analysis Reveals Novel TARs and Prevalent Antisense Transcription in Human Cell Lines
Source: PLoS One. 2010 Mar 25;5(3):e9762. doi: 10.1371/journal.pone.0009762 (PMC2845605; doi:10.1371/journal.pone.0009762)

## Sense overlap

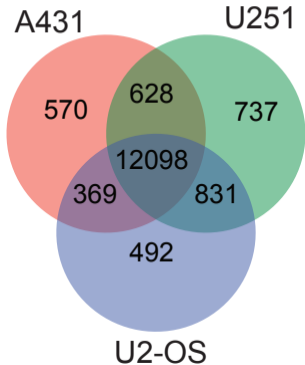

## Antisense overlap

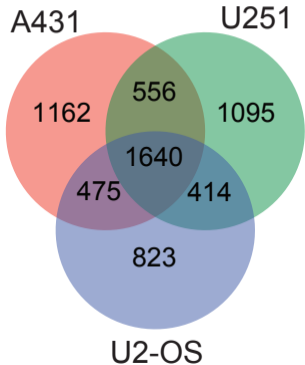

Supplement: Figure S1 — Overlap of sense and antisense expression between the cell lines. (0.23 MB PDF) [file pone.0009762.s001.pdf]

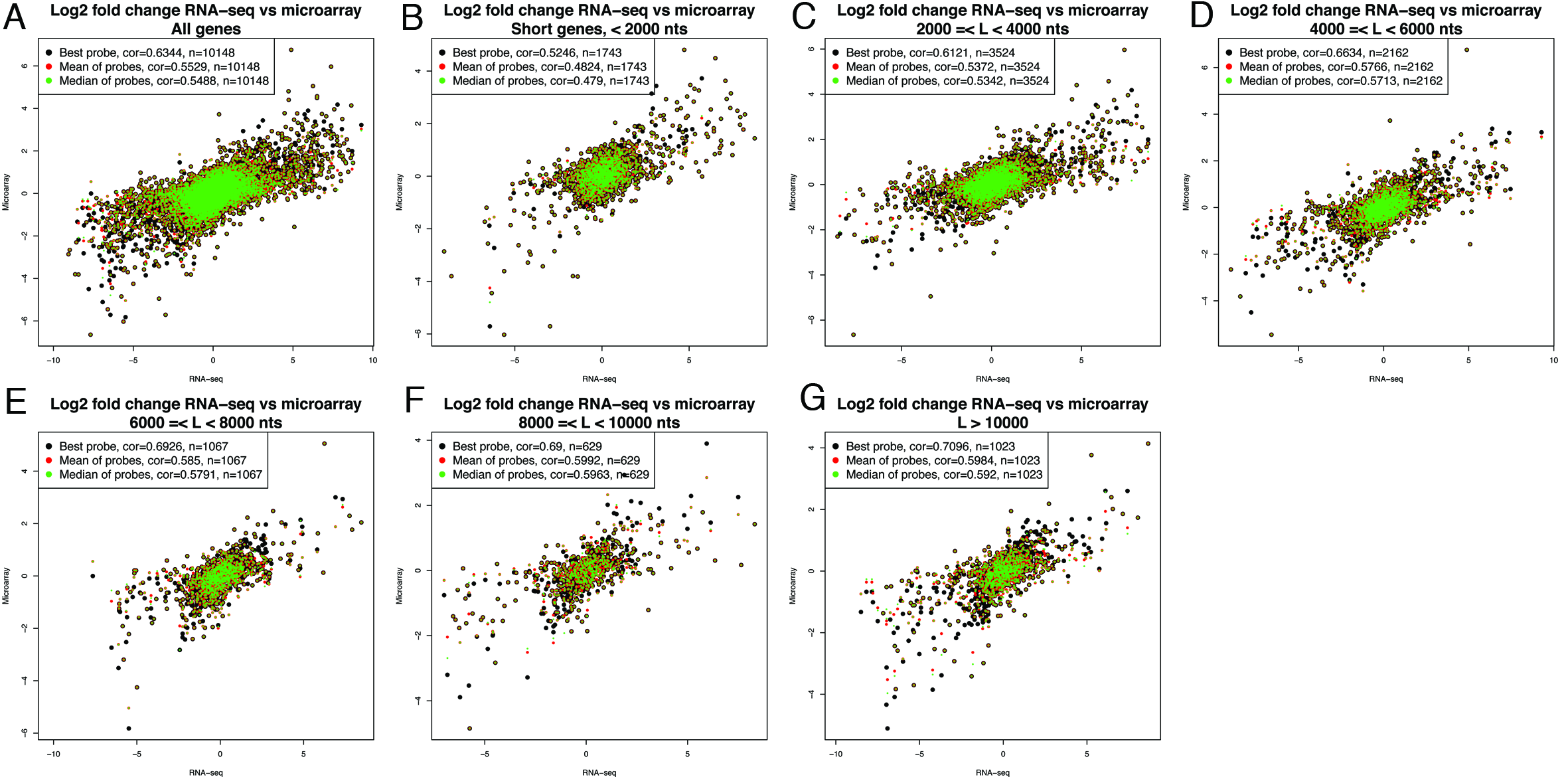

Supplement: Figure S2 — Correlation to microarray data, binned per gene length in intervals of 2000 bps. See main text for discussion. (11.70 MB TIF) [file pone.0009762.s002.tif]

## Sense expression

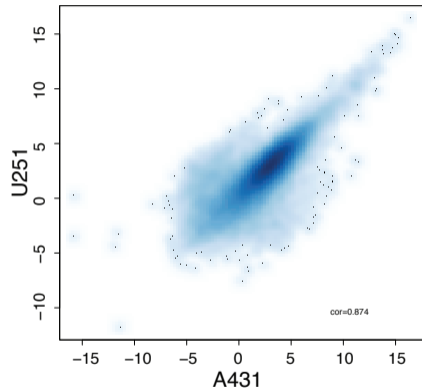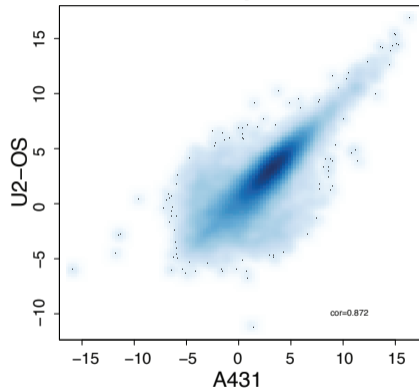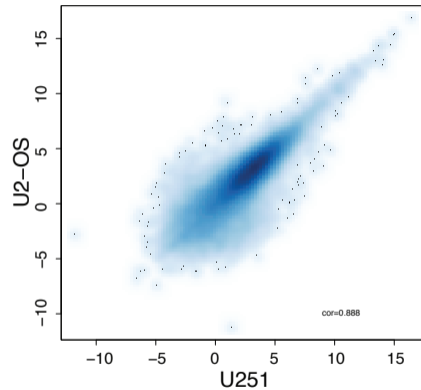

Supplement: Figure S4 — Smooth scatterplots of log10(rpkm) between samples along with Spearman's rho correlation. The correlation is .87 to .88 between all samples. This indicates that most genes have similar levels across all samples. (1.41 MB PDF) [file pone.0009762.s004.pdf]

## Antisense expression

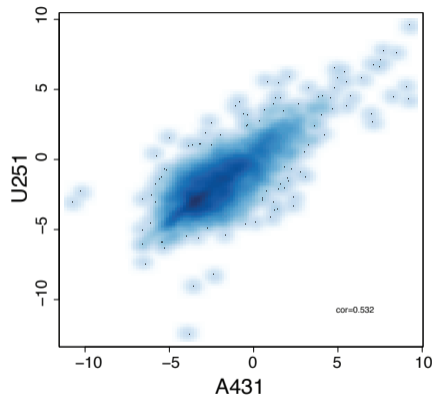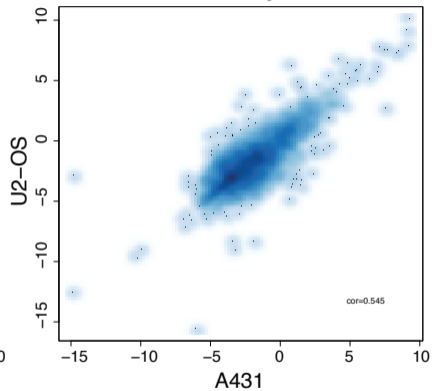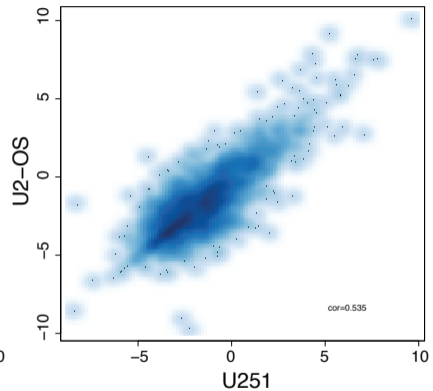

Supplement: Figure S5 — Smooth scatterplots of log10(antisense-rpkm) between samples. Spearman's rho correlation coefficient is here slightly lower than that in the sense-case (supplementary figure S4). A reason for this could be that the majority of antisense transcripts are lowly expressed. It is also possible that these antisense transcripts have regulatory function and differ more than the bulk of mRNAs expressed in a cell. (1.41 MB PDF) [file pone.0009762.s005.pdf]
