# Supplementary material for: In-Depth Transcriptome Analysis Reveals Novel TARs and Prevalent Antisense Transcription in Human Cell Lines
Source: PLoS One. 2010 Mar 25;5(3):e9762. doi: 10.1371/journal.pone.0009762 (PMC2845605; doi:10.1371/journal.pone.0009762)
Supplement: Figure S3 — Information on read mappings for additional cell lines. (D, G) Fraction of reads mapping to different regions. (E, H) Relative tag density in different regions. (F, I) Fraction reads mapping to the sense and antisense strand for different regions. See main text for discussion. (0.20 MB PDF) [file pone.0009762.s003.pdf]

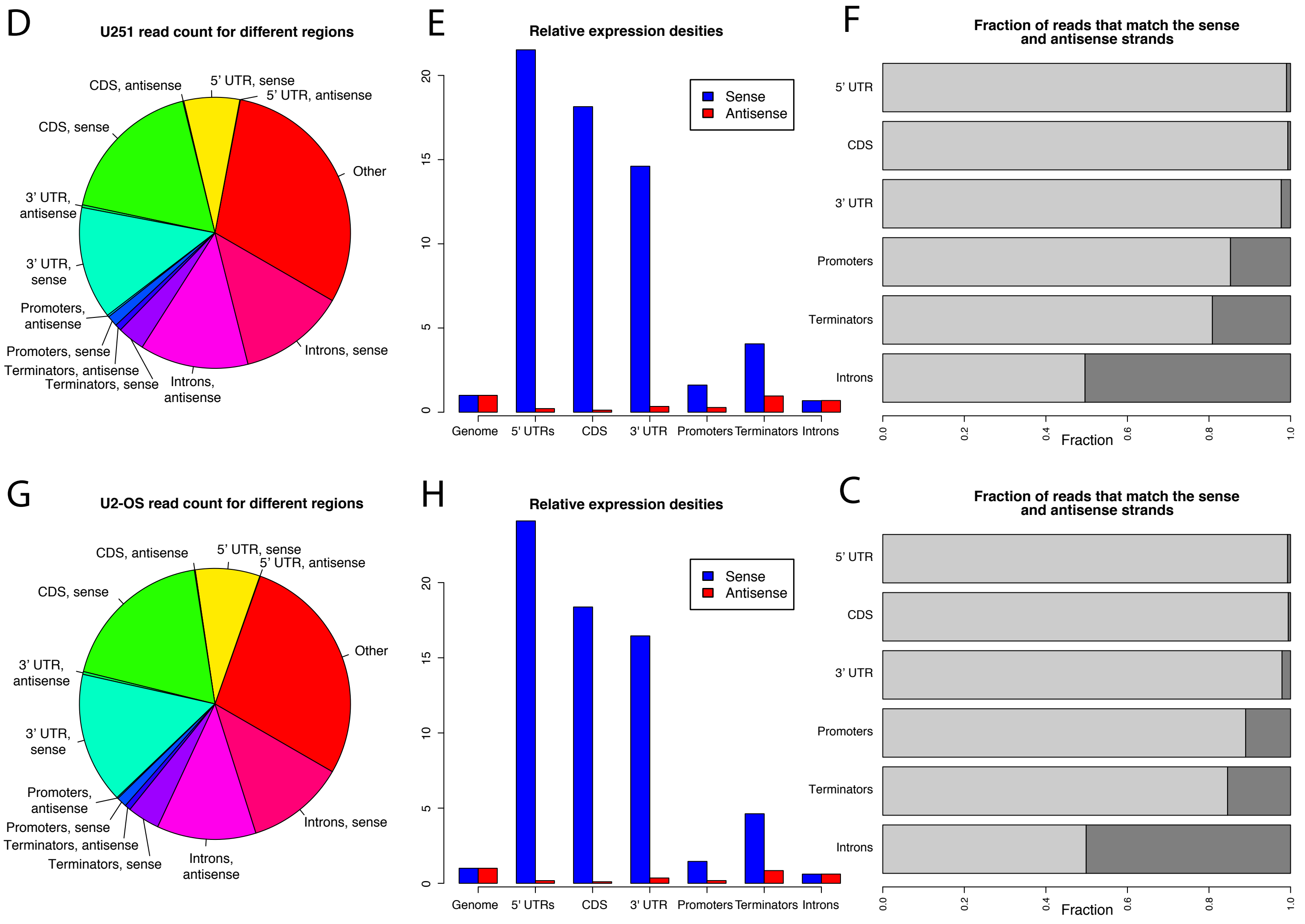
D
U251 read count for different regions
CDS, antisense
5’ UTR, sense
5’ UTR, antisense
CDS, sense
Other
3’ UTR, antisense
3’ UTR, sense
Promoters, antisense
Promoters, sense
Terminators, antisense
Terminators, sense
Introns, antisense
Introns, sense
E
Relative expression desities
Sense
Antisense
0
5
10
15
20
Genome
5' UTRs
CDS
3' UTR
Promoters
Terminators
Introns
F
Fraction of reads that match the sense and antisense strands
5’ UTR
CDS
3’ UTR
Promoters
Terminators
Introns
0.0
0.2
0.4
0.6
0.8
1.0
Fraction
G
U2-OS read count for different regions
CDS, antisense
5’ UTR, sense
5’ UTR, antisense
CDS, sense
Other
3’ UTR, antisense
3’ UTR, sense
Promoters, antisense
Promoters, sense
Terminators, antisense
Terminators, sense
Introns, antisense
Introns, sense
H
Relative expression desities
Sense
Antisense
0
5
10
15
20
Genome
5' UTRs
CDS
3' UTR
Promoters
Terminators
Introns
C
Fraction of reads that match the sense and antisense strands
5’ UTR
CDS
3’ UTR
Promoters
Terminators
Introns
0.0
0.2
0.4
0.6
0.8
1.0
Fraction
